# Mental health status of workers in the semiconductor industry during the COVID-19 pandemic: comparisons between regions on three continents

**DOI:** 10.3389/fpubh.2026.1823067

**Published:** 2026-06-17

**Authors:** Lisbeth Weitensfelder, Michael Kundi, Siegfried Knasmueller, Armen Nersesyan, Michael Schneider, Georg Wultsch

**Affiliations:** 1 Department of Environmental Health, Center for Public Health, Medical University of Vienna, Vienna, Austria; 2 Center for Cancer Research, Medical University of Vienna, Vienna, Austria; 3 Department for Psychiatry and Psychotherapy 2, LKH Graz II, Graz, Austria; 4 asu-experts GesbR, Graz, Austria

**Keywords:** COVID-19, home office, mental health, perceived danger, remote work

## Abstract

**Background:**

The COVID-19 pandemic had detrimental effects on mental health globally, both in and outside the work context. The type of work and work organization additionally had adverse or protective effects on mental health, with sometimes inconsistent findings, like a dualistic effect of working from home. Country differences in mental health effects of the pandemic have been reported, but comparative studies in the work context face the challenge of different work types and different experimental designs. Thus, the present investigation compares connections regarding mental-health status, COVID-19 related experiences and management satisfaction at the workplace with an identical questionnaire design in the same sector between three continents.

**Material and methods:**

1,946 workers from three continents in the semiconductor industry were investigated using validated mental health questionnaires. Additionally, COVID-19-related experiences, organizational factors and satisfaction with SARS-CoV-19 management at the workplace were assessed.

**Results:**

Significant differences between continents could be found regarding mental health parameters, with higher anxiety and lower self-efficacy, but also higher well-being in the Asian group. Significant continent differences could further be found regarding personal experiences with COVID-19, estimated danger and subjective protection against an infection. Working from home was related to most of the assessed mental health parameters in a beneficial way, while at the same time being negatively related to work satisfaction.

**Conclusion:**

Continent-specific differences in pandemic mental health effects should be considered in research, but also in the planning of counseling offers. Working from home might show beneficial mental health effects when implemented correctly.

## 1 Introduction

The impact of COVID-19 on mental health parameters has been investigated in a large number of studies. As of February 2026, nearly 35,700 PubMed-entries dealt with

the topic as central, i.e., it is mentioned in abstract and/or title of the research, with nearly 4,000 of the entries still being published in 2025. Overall, the pandemic led to a higher prevalence of mental health problems, though variations between different timepoints of the pandemic existed (1). The studies mostly deal with two types of questions, namely: (i) how the infection itself affects mental health in the short and long run (including Post and Long COVID) and (ii) how indirect/connected factors (e.g., feelings of anxiety, measures to control its spread, pandemic-related increases of work strain) affect the mental health status.

When it comes to indirect mental health effects, research covered either the general population (including specific age groups such as children or people over 65) and/or specific occupational groups at increased risks such as healthcare workers and teachers. Only very few other workplace-settings were investigated, for example taxi drivers, seafarers, and dentists. The results show that demographic factors, the social status as well as private parameters (e.g., family life) have strong impact on the extent of adverse effects [e.g., Benatov et al. (2)]. Also measures taken to reduce the viral spread are sometimes mentioned as playing a crucial role for mental health. Some single studies showed adverse mental health effects of lockdowns especially with high loneliness and high perceived stress [e.g., Li et al. (3)]. Already studies from the first months of the pandemic showed that countermeasures such as home confinements had adverse effect on mental well-being, with increased reported depressive symptoms (4), and negative effects on life satisfaction and social participation (5). But not all reviews dealing with mental health consequences of lockdowns reported a lockdown impact as equally strong or worrisome [e.g., Prati and Mancini (6) vs. Ganesan et al. (7)], and stated that adverse effects on mental health might also not always be visible (immediately): Hospital admissions for suicide attempts for example were even lower during lockdowns than after (8). Overall though, the pandemic had negative effects on suicidality: A systematic review concluded that regardless of a stable suicide rate during the pandemic, suicidal ideation and suicide attempts increased (9). Another systematic review regarding suicidal trends during the pandemic reported associated risk factors such as financial crises, quarantine and social distancing (10). Thus, while lockdowns and home confinement policies may have contributed to negative mental health outcomes, they are intertwined with social and personal variables that additionally contributed to mental health outcomes. For example, an English study showed that those individuals who applied to home confinement even in times of eased governmental containment measures had a worse mental health outcome (11). An international study from the early phase of the pandemic covering 78 countries and containing nearly 10,000 adults concluded that social support, education, psychological flexibility, finances and access to necessities were consistently connected to mental health during lockdowns (12). Some of these factors—for example finances, but also social participation—are related to work life, and indeed a review from the first year of the pandemic concluded that work and organizational interventions can mitigate adverse mental health outcomes (13), and perceived income loss was associated with adverse mental health conditions (14).

Interestingly, also resuming to work and easing restrictions were associated with adverse psychological reactions, showing symptoms like distress, somatization or emotional reactions, with strong evidence that deficiency of workplace measures is associated with higher stress (15). Mental issues connected to the workplace during the pandemic did not affect all types of work equally, being worse for healthcare workers on the frontline, migrants and public workers (13). The different effect on different work types puts the spotlight on work-related factors, showing that workplace organization and work conditions are associated with a variety of psychosocial risks (16) and chances. For example the possibility to work from home has shown to be associated with both benefits (such as freedom and flexibility) as well as downsides such as loneliness or depression (17, 18). A German study investigating the first lockdown showed that working from home was associated with a small negative effect on mental health, particularly regarding loss of interest and loneliness (19), but the study focused on the lockdown period with overall little social interaction. Overall, the relationship between working from home and mental health seems complex with no clear association respective a dualistic one (17, 20).

While (social and work) environments might represent supporting or protecting factors for employees' mental health, also individual factors play a crucial role in order to deal with crises. In terms of the pandemic, coping skills showed to play an important role in sustaining the mental health of healthcare workers, whereas avoidant coping styles were associated with increased stress (21). Also self-efficacy (22), which represents the individual's believe in being able to produce a certain aspired outcome, seems to be important for resilient behavior (23). In the context of the pandemic, resilience factors seem to be negatively connected to psychological distress, both in the general population as well as in healthcare workers and patients (24). However, the quality of resilience studies regarding the pandemic is also criticized as not being sufficient in order to draw overall conclusions (25, 26).

Detrimental effects of the pandemic on mental health was observed globally (27, 28), though with country differences. Country differences can also be found in the political measures to control the spread of the virus (29), but also in factors such as SARS-CoV-2 seroprevalence rates (30) or regarding the countries' (pre-pandemic) prevalence of mental disorders, with pronounced differences regarding psychological parameters such as anxiety or depression (28), and on a continental level also regarding obsessive compulsory behavior (31). A study of the COVID-19 Mental Disorder Collaborators (28) suggested that the locations where the pandemic hit hardest in terms of decreased mobility and infection rate also had the greatest increase in depression and anxiety disorders.

Differences regarding mental health consequences also exist regarding its research coverage. The biggest proportion of early publications regarding mental health effects of the pandemic comes from the USA, followed by China (32), while in a later analysis most studies derive from China, followed by the USA (33). Comparative studies and meta-analyses are available for single regions or continents such as Central and Eastern Europe (2, 34–38), Africa (39, 40) and Southeast Asia (41, 42). Comparisons between continents base on meta-analyses (31, 43–46) which have the disadvantage that the individual studies differ in their experimental design.

The present study describes mental health parameters of workers in the semiconductor industry during the pandemic, all assessed with the same measurement instruments. Participants included individuals who are involved in the production of semiconductors as well as administrative staff, with manual workers from two European countries (Austria and Germany) and non-manual workers from these two countries plus 11 more. No investigations with workers from the electronic industry has been realized during the COVID-19 pandemic according to our knowledge, though a German study examined mental health impacts of the early pandemic on the automotive industry (47). In the electronic industry, Sznajder et al. (48) examined occupational stress and mental health among Chinese women factory workers before the pandemic. They found that high job strain was associated with hopelessness, not feeling useful and feeling depressed.

The information for the present study was collected by use of established questionnaires from approx. 2,000 participants from 7 countries in Europe, 5 countries in Asia and the USA. Furthermore, individual COVID-19-associated parameters were analyzed by use of a self-designed questionnaire. The results which we obtained were used to conduct systematic comparisons of mental health parameters in workers in the three continents. While our study does not aim to be representative of the included continents, it should provide information whether mental health outcomes are comparable across different global regions. For language simplicity, we use the term “continents”. In addition, we analyzed the impact of individual factors on mental health parameters and on satisfaction with SARS-CoV-2 resp. COVID-19 specific measurements in multivariate analyses.

## 2 Materials and methods

### 2.1 Research context

The survey was conducted as a mental health initiative of the company in order to focus on possible mental health problems during the ongoing COVID-19 pandemic and underwent company-intern evaluation via data protection commission, gender equality office and works council. Staff members were informed via the CEOs, mental health advisors, occupational physicians, and employee representatives. The participation via online survey was strictly anonymous and happened via single-use codes and after up to two e-mail reminders.

### 2.2 Participants

The study was realized between January and February of 2022. In total 7,174 individuals from 13 countries (5 from Asia, 7 from Europe, and the USA) were invited to participate, with widely differing employment numbers in each country. An overview is listed in Table 1. Participation rates varied from country to country, with the highest participation rate being in the USA (222 out of 277 invited employees) and the lowest rate being in China (45 out of 521 invited employees; an overview regarding total amounts and participation rates can be found in the supplementary material, Table S1). Thousand nine hundred sixty-one (27.3%) individuals completed the survey, with 1,946 reporting their gender as male or female and thus being included in analyses that required gender. Five hundred ninety-three (30.5%) employees of this resulting sample had executive functions. The respective numbers of participants from each continent are listed in Table 1, additionally listing countries and the respective governmental response to the pandemic in a single aggregated variable. Overall, the majority of participants were males: The highest amount of females was in Asia (36.5%), while percentages of females in Europe (33.2%) and the USA (30.6%) were insignificantly lower. Age was assessed in four categories, showing a higher mean age in the USA. Additional survey data overview can be found in the supplementary material (Table S2).

**Table 1:** Numbers of participants per country and continent.

Continent	Country	Mean governmental response index 2020–2021^*^	*n*	Age ≤ 35 years (%)	Age > 45 years (%)	Female (%)	No home office (%)
Asia	China	67.94	20 ≤ *n* < 50
	India	61.82	20 ≤ *n* < 50
	Malaysia	65.16	*n* > 500
	Philippines	61.0	20 ≤ *n* < 50
	Singapore	62.82	50 ≤ *n* < 100
	Overall Asia	64.73^a^	695	21.2^c^	34.5^c^	36.5^c^	10.6^c^
Europe	Austria	62.59	>300
	Czech Republic	56.28	< 20
	Germany	55.49	>500
	Italy	67.22	20 ≤ *n* < 50
	Slovakia	59.07	20 ≤ *n* < 50
	Switzerland	49.77	20 ≤ *n* < 50
	UK	59.43	< 20
	Overall Europe	57.97^a^	1,032	26.5^c^	39.4^c^	33.2^c^	28.7^c^
North America	USA	55.55^b^	219	18.3^c^	63.5^c^	30.6^c^	33.8^c^

Analyses base on continent level, categorical country data displayed for information.

^*^The governmental response index represents pandemic response measures by the countries' governments; it is an aggregated variable consisting of four areas (containment and closure policies, economic policies, health system policies and vaccination policies), possibly ranging between 0 and 100. It indicates on a daily basis how many policy types a government has acted upon, regardless of the actions' effectiveness. Mean government response indices were calculated from daily values for the periods of January 1st, 2020, until December 31st, 2021, using data from the Government Response Tracker (CC BY 4.0 License), Blavatnik School of Government, University of Oxford (Hale et al., 2021), see also (29).

^a^Continent-specific weighted mean values based on included countries in our study, with weights proportional to the number of participants in each included country.

^b^USA without Virgin Islands.

^c^Pearson Chi-Square values regarding continent comparisons significant for age (combined to 3 groups: up to 35 years, 36–45 years, 46+ years) and home office (dichotomized: yes/no), but not for gender (male/female). Age: Pearson Chi-Square = 76.512, df = 4, *p* < 0.001. Home office: Pearson Chi-Square = 92.552, df = 2, *p* < 0.001. Gender: Pearson Chi-Square = 3.849, df = 2, *p* = 0.146.

### 2.3 Measures

Personal demographic and COVID-19-specific data were collected with a self-administered questionnaire. The questionnaire consisted of demographics and work place characteristics, experience with COVID-19 (two items: experience of a relative infected with SARS-CoV-2, and experience of a COVID-19 death in relative; both questions dichotomous) and attitudes toward the company's management of the COVID-19 crisis and estimated danger. These assessed attitudes covered the following topics, each with one item at a 5-point Likert scale: (i) Work satisfaction (“very satisfied” to “very unsatisfied”), (ii) COVID-19 info satisfaction (“very satisfied” to “very unsatisfied”), (iii) COVID-19 management satisfaction (“very satisfied” to “very unsatisfied”), (iv) feeling protected at the workplace (“yes” to “no”), (v) sufficiency of protection equipment at the workplace (“yes” to “no”), (vi) sufficiency of hygiene measurements (“yes” to “no”), (vii) support by superiors (“yes” to “no”), (viii) fear of contracting COVID-19 (“yes” to “no”), and (ix) feeling endangered by the virus (“yes” to “no”).

Furthermore, all participants were asked to fill out standardized forms that provide information about several mental health parameters: (i) self-efficacy was measured with General Self-Efficacy Scale (GSE) (49) which consists of 10 items and was designed to measure the general believe of an individual to deal with a broad range of stressful or challenging demands; (ii) the SF-12 health survey questionnaire contains 12 items as measure of physical and mental health (50, 51); (iii) the WHO-5 Well Being Index is the most widely used questionnaire assessing subjective well-being (52); (iv) depression was assessed by use of the Depression Anxiety Stress Scale 21 (DASS-21) which was designed to detect the distinct features of depression, anxiety and stress (53, 54); (v) the Insomnia Severity Index (ISI) developed by Morin (55) is a reliable and valid instrument to quantify insomnia (56) and is currently one of the most frequently used insomnia questionnaires. It is a seven item instrument to assess sleeping problems (insomnia) based on the criteria of the International Classification of Sleep Disorders (57).

### 2.4 Statistical analyses

To account for (significant and unsignificant) differences in demographic parameters across regions, all analyses were adjusted for age and gender by inclusion of these variables into the multivariate model and reporting the parameter estimates adjusted for these covariates. Distribution analysis of the variables (scores and scales) except results obtained with the WHO-5 Well-Being Index showed significant deviations from normality and were McCall (area) transformed (58, 59) except the ISI score that was log-transformed. For the presentation of the results in graphs, the values were back-transformed to represent the original scale; values *p* < 0.05 were considered statistically significant.

To assess the impact of social-demographic personal and physiological factors on mental health parameters and on satisfaction with SARS-CoV-2- and COVID-19-specific measures, multivariate logistic regression analyses were performed in which one category was selected as a “reference group” (also called a “base category”). The coefficients describe how the independent variables are related to the probability of being in a specific outcome group vs. the reference group.

## 3 Results

All raw scores (untransformed) of the applied questionnaires were correlated (Spearman), with no correlation coefficient reaching values of 0.80 or above. Self-efficacy showed small (*r* < 0.3) positive correlations with reported health quality of life (SF-12) and well-being (WHO-5) as well as small negative correlations with depression, stress, anxiety (DASS-21) and insomnia (ISI). Well-being (WHO-5) was additionally positively correlated with physical and mental health quality of life in the SF-12 (0.27 and 0.66) as well as negatively correlated (−0.49 to −0.71) with depression, anxiety, stress and insomnia. Correlation coefficients of the DASS-21 scales, insomnia and mental health quality of life (for the latter negatively) ranged between 0.62 and 0.79. An overview regarding correlation coefficients is listed in Table 2.

**Table 2:** Spearman correlation matrix of questionnaire raw scores.

	1	2	3	4	5	6	7	8
1. Self-efficacy (GSE)	1	0.163	0.239	0.228	−0.218	−0.276	−0.218	−0.189
2. Physical health (SF-12)		1	−0.163	0.266	−0.300	−0.364	−0.295	−0.402
3. Mental health (SF-12)			1	0.657	−0.722	−0.568	−0.670	−0.608
4. Well-being (WHO-5)				1	−0.705	−0.492	−0.670	−0.593
5. Depression (DASS-21)					1	0.682	0.795	0.621
6. Anxiety (DASS-21)						1	0.700	0.563
7. Stress (DASS-21)							1	0.622
8. Insomnia (ISI)								1

*n* = 1,946, all *p*-levels < 0.001, two-sided.

### 3.1 Comparison between continents regarding mental health parameters

**Figure 1:**
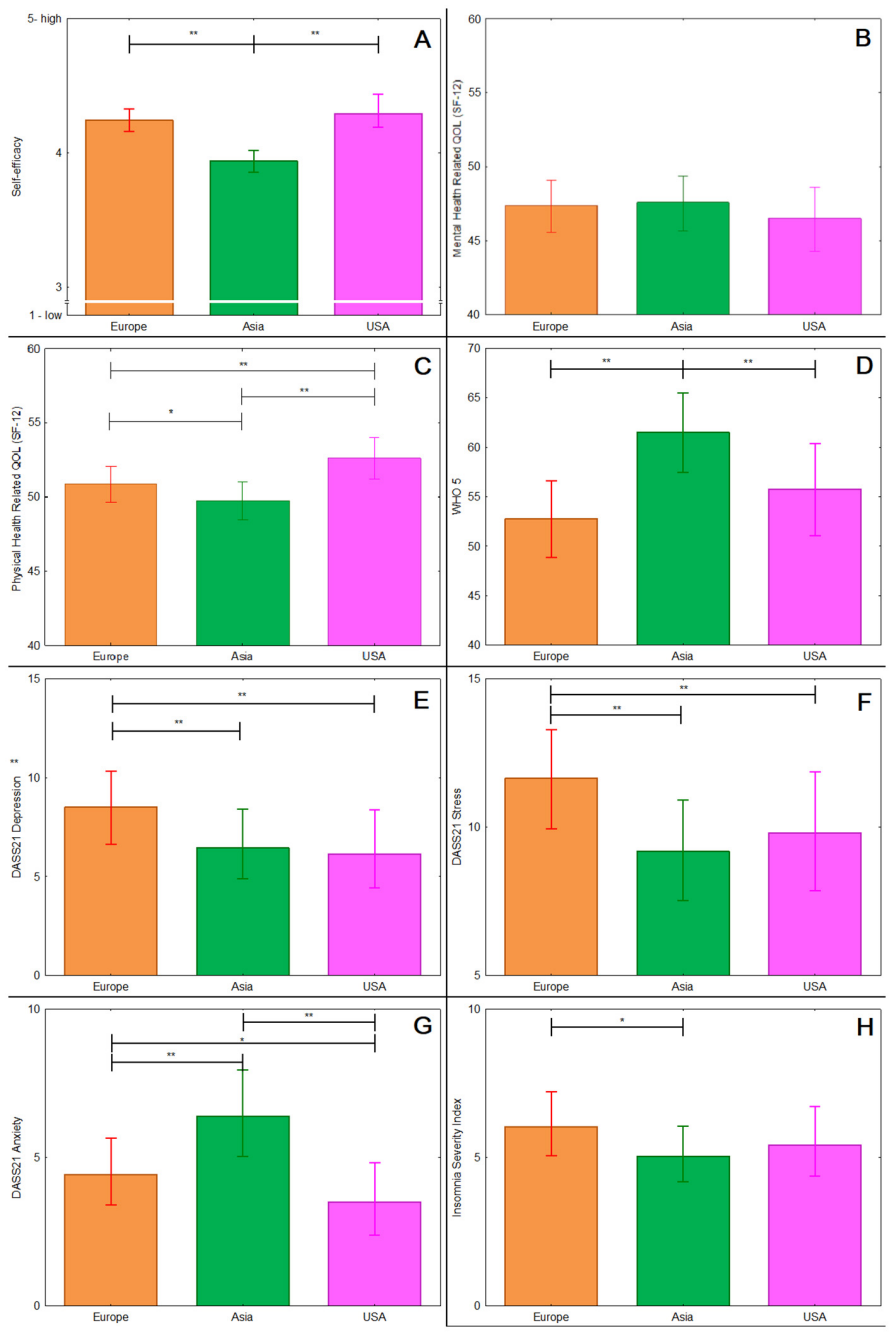
**(A–H)** Results of the evaluation of the different questionnaires. Adjusted means are shown as bars, whiskers represent 95% *CI*. Stars indicate significance (*p* ≤ 0.05). The values on the y-axis represent the respective questionnaire score.

**Figure 2:**
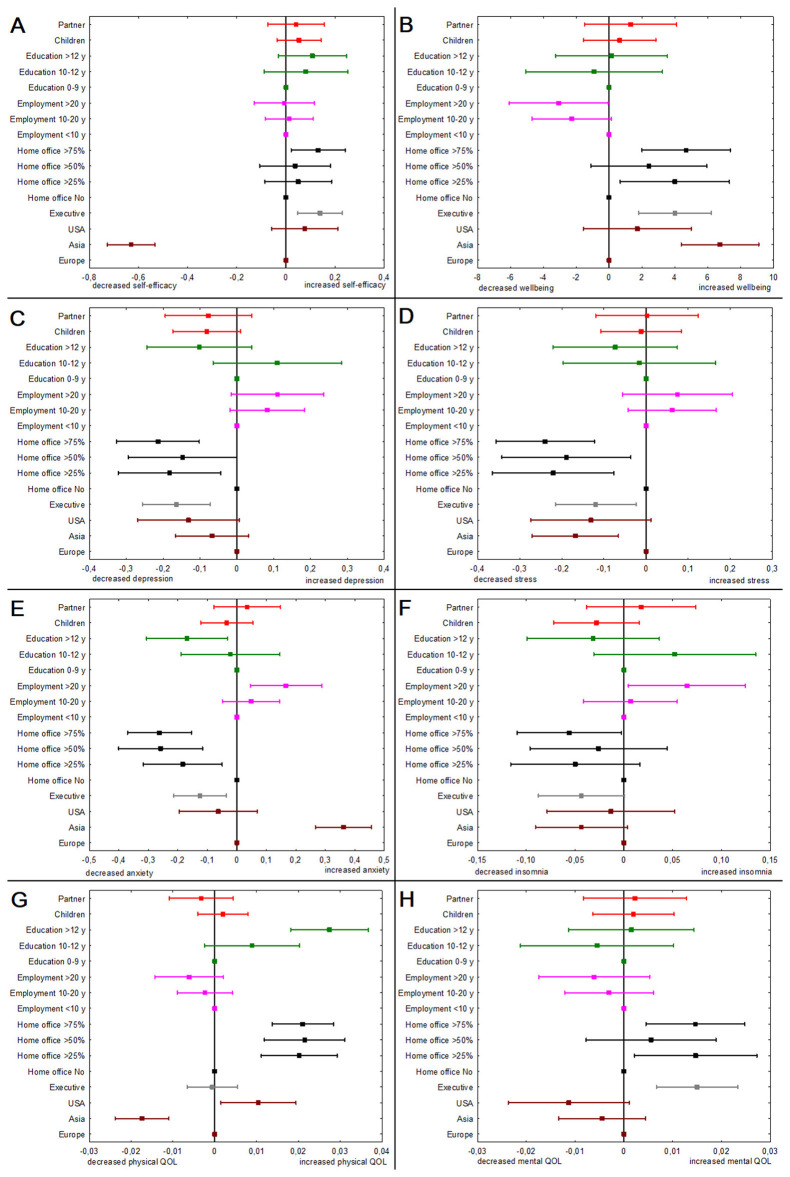
Impact of individual parameters on mental health, showing the decreasing (left part of each graph) to increasing (right part of each graph) influence of the reported variables on self-efficacy **(A)**, well-being **(B)**, depression **(C)**, stress **(D)**, anxiety **(E)**, insomnia **(F)**, physical health quality of life **(G)** and mental health quality of life **(H)**. In case of multicategorical predictors, one category is selected as reference and the impact of the other categories must be interpreted relative to the reference category. Dichotomous predictors are displayed as yes vs. no. Points indicate regression coefficients, whiskers their 95% confidence interval. All analyses are adjusted for gender and age.

Figures 1A–2H summarize the results of comparisons between the different continents.

The highest extent of self-efficacy was measured in the USA and the lowest value was recorded in Asia (Figure 1A). In the USA and in Europe significantly higher values were found (15% and 11%, respectively) compared with Asia. No significant difference could be detected between Europe and the USA (*p* = 0.154).

No difference was found between the continents in regard to mental health related quality of life (Figure 1B). Regarding physical health related quality of life (SF-12 health survey questionnaire) all continents differed from each other substantially. Notably, participants from the USA reached the highest scores for physical health (Figure 1C).

The highest well-being indices were found in participants from Asia. The USA and Europe had lower values which did not differ significantly from each other (*p* = 0.065) while Asians had distinctly higher values as the other continents (*p* < 0.001) (Figure 1D).

The highest depression scores were recorded in Europe; i.e., this parameter was 24% higher than in Asia and 27% higher than in the USA (Figure 1E). The difference between Asia and Europe (*p* = 0.002) as well as between the USA and Europe (*p* < 0.001) were statistically significant, while the USA and Asia had similar values (*p* = 0.707). The stress indices showed a more or less identical pattern, i.e., the highest values were recorded in Europe while the Asian participants had the lowest scores (Figure 1F). The differences to Europe were highly significant (Asia *p* < 0.001, USA *p* = 0.010). The values between Asia and the USA were similar (*p* = 0.373). Anxiety levels were highest in Asia (31% higher than in Europe and 43% higher than in the US; *p* < 0.001 in both cases; Figure 1G). It is notable that we found significant differences in anxiety between all continents. As for sleeping problems, assessed via insomnia severity index (ISI), the highest score was found Europe and the lowest in Asia (Figure 1H). Significant differences however were only found between Europe and Asia (*p* < 0.001). The differences in insomnia between the USA and Europe and between Asia and the USA were not significant (*p* = 0.151 and *p* = 0.345, respectively). Severity of mental health effects as well as means adjusted for age and gender are displayed in the Supplementary material (Tables S3, S4).

### 3.2 Estimated danger of COVID-19 and COVID-19 experiences

Aside from the reported continent differences in individual variables, personal experience with COVID19 cases varied widely too: Experience of a relative infected with COVID-19 was reported by 83% in the US, followed by 70% in Europe and 45% in Asia; all differences between regions were highly significant (*p* < 0.001). Interestingly not the same differences were seen concerning the experience of a COVID-19 death in a relative. Although highest proportions were again reported in the USA (25%), these values were not significantly different from the 20% reporting a death in relatives in Asia. Europe had 11% such experiences reported, a value highly significantly (*p* < 0.001) lower as compared to both Asia and the USA. A graphical illustration of this result can be found in the Supplementary materials (Figure S1).

On continent level, also significant differences regarding fear of getting infected or estimated danger of getting infected could be found: In both variables Asians reported much higher values (p < 0.001) than Europeans or workers from the USA. European participants expressed least fear of getting infected or less danger also compared to the USA (*p* < 0.001). A graphical illustration of these results can be found in the Supplementary materials (Figure S2).

As continent specific analyses alone do not reveal the complexity of determinants including personal and psychological influences, the following results contain multivariate analyses with inclusions of personal parameters, socio-demographic factors and psychological determinants.

### 3.3 Impact of individual variables on mental health parameters

The reported scores in the psychological scales show dependencies on socio-demographic attributes. In the following (Figure 2), results of multiple linear regressions are reported. The strength of the influence of certain variables in positive and negative sense is graphically presented (together with 95% confidence intervals). Aside from continent differences, especially the amount of home office showed a significant influence on several parameters associated with mental health (DASS-21 scores), but also with physical quality of life (SF-21). Detailed numerical results (adjusted regression coefficients and *p*-values) are displayed in Table S5 in the Supplementary materials.

### 3.4 Impact of individual variables on occupational parameters and perception of pandemic danger

**Figure 3:**
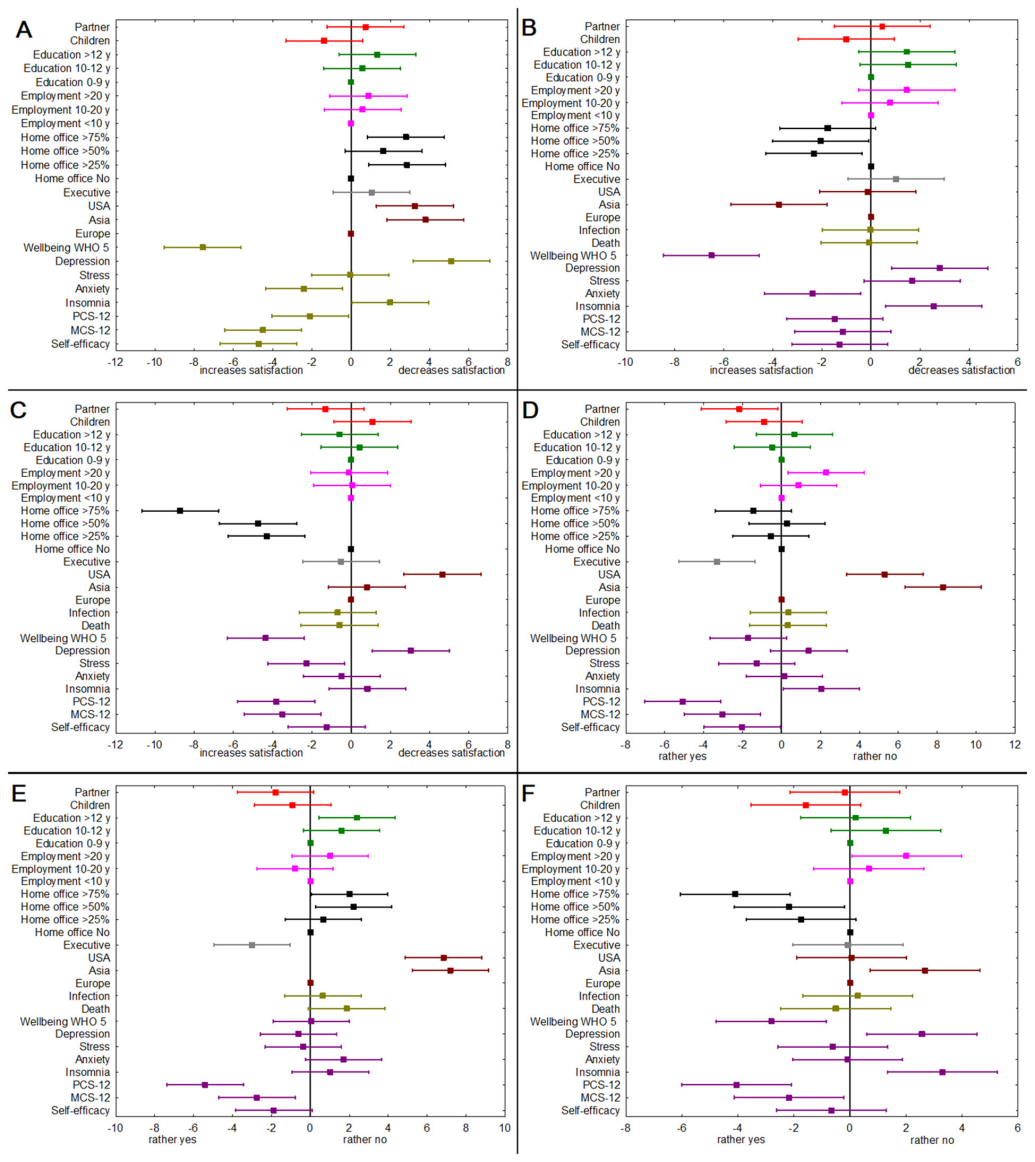
**(A–F)** Results of multivariate analyses (graphical illustrations of ordered logistic regressions) regarding work satisfaction **(A)**, COVID-19 info satisfaction **(B)**, satisfaction with COVID-19 management **(C)**, feeling protected against an infection **(D)**, sufficient equipment for protection **(E)**, and assessment of management support **(F)**. PCS-12 = Physical health quality of life (SF-12), MCS-12 = Mental health quality of life (SF-12). Dichotomous predictors are displayed as yes vs. no. Points indicate regression coefficients, whiskers their 95% confidence interval. For multicategorical predictors one category is selected as reference and the impact of the other categories must be interpreted relative to the reference category. All analyses are adjusted for gender and age.

Personal parameters of the responders to management activities and measures in response to the COVID-19 pandemic were analyzed in a multivariate ordered logistic regression analysis. The results are shown graphically in Figures 3A–F.

Variables which affect work satisfaction are specified in Figure 3A. Family life, children, the education level and duration of employment had no significant impact while working in home office had a negative impact. Participants from the USA and Asia had higher scores than those from Europe. Stress had no clear impact while well-being as well as physical and mental health and self-efficiency increased the work satisfaction while depression and insomnia decreased the work satisfaction.

Most personal variables had no strong impact on the satisfaction with COVID-19 information (Figure 3B). Participants from Asia were more satisfied than participants from the other continents. Among the physiological factors anxiety and insomnia had no impact, high scores of well-being (WHO-5) as well as physical and mental fitness and stress correlated positively, while depression decreased the extent of satisfaction. In regard to satisfaction with Covid-19 management (Figure 3C) living with a partner or children did not have an influence. Also education and duration of work in the company did not significantly contribute. Participants from the USA had lower satisfaction as compared with Europeans.

It can be seen in Figure 3D that family life improved the feeling to be protected against infection. Participants from the USA and Asia felt less protected. High levels of physical and mental fitness and self-efficacy were positively associated with a higher feeling of protection while insomnia was negatively associated with feeling protected.

Two further variables which were analyzed concerned the satisfaction with provision of protective equipment and hygienic measures at the work place. We found a very similar pattern of both parameters; only the results which were obtained with the first variable are shown graphically (Figure 3E). High physical and mental health scores had a positive impact on the satisfaction level.

The satisfaction with the management support of COVID-19 is shown in Figure 3F. Home office was associated with a higher degree of satisfaction. Insomnia and stress correlated negatively while well-being as well as mental and physical health quality of life were associated with positive statements.

Detailed numerical results are displayed in the Supplementary materials (Table S6).

### 3.5 *Post-hoc* analysis: mental health and pandemic perception in European high vs. low govermental response countries

During the pandemic, government reactions to prevent the spread of the disease differed widely on country level. Mean values calculated from data of the Oxford COVID-19 Government Response Tracker (OxCGRT) (29) show that governmental response overall seemed higher in our Asian sample than in our European one (Table 1), with exception for two countries, namely Austria and Italy. Thus, we separated Europe ex post in a high- (mostly Austria and some cases from Italy) and a low-governmental-response part (including the other European countries in our sample) and compared them regarding their COVID-19 related perceptions, the participants' self-efficacy and their well-being indices. Group comparisons are shown in the Supplementary material (Table S7): Due to existing differences in age groups between the considered European countries, Table S7 also shows mean ranks for age groups separately. Regarding gender, no distribution difference in the considered European subsample could be found (Pearson Chi-Square = 0.259, df 1, *p* = 0.611).

While there are no differences regarding well-being, some COVID-19 related perceptions differ, showing less fear of COVID-19 and less perceived danger in the high-governmental-response countries. A closer look shows these differences are limited to the age group of up to 35 years. All other differences become insignificant when controlling for multiple testing (Bonferroni).

## 4 Discussion

To frame this discussion appropriately, it has to be noted that our results are not generalizable for continents overall with their complex cultures and vast heterogeneity. Our investigation bases on a very specific industry with several limitations. For simplified wording we use the term “continents” when comparing results from participants of different global regions.

Looking at results of our world-wide survey regarding mental health parameters during the COVID-19 pandemic, we found significant differences between Asia, Europe and the USA. Overall, participants from Asia reported higher anxiety and lower self-efficacy but also higher well-being, while physical health-related quality of life was estimated highest in the USA and lowest in Asia. Differences were also observed in perceived protection against infection and sufficiency of protective equipment, with participants from Asia and the USA feeling less protected.

Three main aspects of the results should be highlighted, which are associations between individual variables and well-being, continental differences in the perception of COVID-19 risk, and the role of organizational factors.

In our cross-sectional design, organizational factors, particularly home office arrangements, were positively associated with several mental health indicators. Working from home was linked to increased well-being, reduced stress, depression and anxiety, and higher health-related quality of life. Interestingly, home office was also associated with higher physical health quality of life and greater satisfaction with COVID-19 management. However, it was negatively associated with work satisfaction. Previous studies suggest that support from coworkers and supervisors is a determinant of job satisfaction (60, 61). Reduced interpersonal interaction and feedback during remote work may therefore contribute to lower work satisfaction. In addition, workplace relationships represent an important source of social connections after the home environment (62). The benefits of working from home may also depend strongly on the home-office environment itself (63). Consequently, remote work policies should be implemented alongside strategies that maintain social interaction and organizational support. Flexible work arrangements have been shown to improve well-being during the pandemic, for example among working mothers (64).

Regarding continent comparisons, an interesting pattern emerged: Self-efficacy is typically positively associated with mental health and well-being (49, 65, 66), which could also be confirmed via small correlations in our overall dataset. But in country comparisons, Asian participants reported lower self-efficacy and higher anxiety but still higher well-being compared with participants from Europe and the USA. Several explanations may account for this finding. First, well-being is influenced by many factors (67), and our study assessed only a subset of these. Second, cross-cultural differences in questionnaire interpretation may exist. Methods such as item response theory and differential item functioning could help determine whether the instruments measure equivalent constructs across cultural contexts. Third, residual confounding may remain despite adjustment for age and gender (68). Though below the threshold for significance, the Asian sample included a higher proportion of women, commonly associated with greater anxiety risk. Additionally, the Asian sample contained less participants in the highest age group, especially compared to the USA, while higher age groups are considered less vulnerable to anxiety. Gender and age differences in self-efficacy and anxiety have been documented both in general as well as in the pandemic context (28, 69–73). Finally, cultural differences in the perception or reporting of well-being may contribute to the observed pattern. These findings underline the importance of considering cultural context when applying psychological constructs in global research.

Perceived danger of COVID-19 infection also differed between continents. Participants from Asia reported a higher perceived infection risk despite reporting fewer infections among relatives (45%) than participants from the USA (83%) or Europe (70%). Nevertheless, deaths among relatives were reported at comparable levels in Asia (20%) and the USA (25%), which may partly explain the higher perceived danger. Differences in governmental responses and risk communication may also influence risk perception. While some pandemic measures triggered public resistance resp. feelings of reactance [e.g., mandatory vaccination debates (74)], our results indicate that home office policies were associated with more favorable mental health outcomes.

Participants from Asia and the USA also reported feeling less protected against infection. Whether stricter governmental responses, overall higher in Asia, contributed to increased anxiety remains unclear. A *post-hoc* comparison within Europe suggested that countries with stronger governmental responses reported lower fear of infection in younger participants; however, such associations cannot be interpreted causally.

Several limitations should be noted. Although surveying employees from the same industry improves comparability, cultural differences in questionnaire interpretation cannot be excluded. For reasons of feasibility, COVID-19 related variables were measured using single items. Cultural differences in interpretation might also apply to them. In addition, employees differ in responsibilities and response rates across countries, and the sample is not representative of the general population. Organizational culture at both industry and regional levels may also influence responses. Furthermore, the governmental response index aggregates diverse policy measures that may affect populations differently. Future studies should therefore examine specific policy measures in greater detail. Finally, the cross-sectional design precludes causal conclusions. Thus, especially in terms of mental health consequences of organizational factors, longitudinal research shall be encouraged.

In conclusion, our findings demonstrate significant continental differences in pandemic-related mental health indicators. Participants from Asia reported higher anxiety and lower physical health quality of life but also higher overall well-being. These differences may reflect both genuine variations in experiences and cultural differences in questionnaire interpretation. Such factors should be considered when applying psychological instruments internationally and when designing psychoeducational interventions. The results also highlight the importance of organizational measures such as home office during the pandemic. Home office was associated with improved mental health indicators and physical health quality of life, although it was negatively related to work satisfaction. Accordingly, remote work policies should include organizational strategies that support social interaction and employee well-being.

## Data Availability

The raw data supporting the conclusions of this article will be made available by the authors, without undue reservation.
