# Supplementary material for: Mental health status of workers in the semiconductor industry during the COVID-19 pandemic: comparisons between regions on three continents
Source: Front Public Health. 2026 Jun 17;14:1823067. doi: 10.3389/fpubh.2026.1823067 (PMC13318752; doi:10.3389/fpubh.2026.1823067)
Supplement: Supplementary file 1 [file Supplementary_file_1.docx]

Supplementary Material

# Supplementary Figures and Tables

## Supplementary Tables

**Table S1 (Supplement).** Participation rates per country.

| **Countries** | **# Employees**  **Invited** | **# Employees participated**  **(Survey completed)** | **% Employees participated**  **(Survey completed)** |
| --- | --- | --- | --- |
| **nonmanual worker** |  |  |  |
| Austria | 421 | 205 | 48.7 |
| China | 521 | 45 | 8.6 |
| Czech Republic | 46 | 15 | 32.6 |
| Germany | 1171 | 479 | 40.9 |
| India | 51 | 18 | 35.3 |
| Italy | 76 | 21 | 27.6 |
| Malaysia | 1070 | 511 | 47.8 |
| Philippines | 171 | 39 | 22.8 |
| Singapore | 623 | 87 | 14.0 |
| Slovakia | 128 | 41 | 32.0 |
| Switzerland | 92 | 38 | 41.3 |
| UK | 64 | 9 | 14.1 |
| USA | 277 | 222 | 80.1 |
| **manual worker** |  |  |  |
| Austria | 338 | 128 | 37.9 |
| Germany | 2125 | 103 | 4.8 |
| **(TOTAL)** | **(7174)** | **(1961)** | **(27.3)** |

*Notes.* Participation rates including participants with diverse gender.

**Table S2 (Supplement).** Demographic survey overview based on continents

|  | **Age** | | **Gender** | | **Tenure** | |
| --- | --- | --- | --- | --- | --- | --- |
|  | **Category** | **N (%)** | **Category** | **N (%)** | **Category** | **N (%)** |
| Europe (n = 1039) | <25 years | 23 (2.2%) | Male | 694 (66.8%) | <10 years | 452 (43.5%) |
|  | 25-35 years | 252 (24.3%) | Female | 338 (32.5%) | 10-20 years | 291 (28.0%) |
|  | 36-45 years | 355 (34.2%) | Diverse | 7 (0.7%) | >20 years | 296 (28.5%) |
|  | >45 years | 409 (39.4%) |  |  |  |  |
| Asia (n = 700) | <25 years | 6 (0.9%) | Male | 441 (63.0%) | <10 years | 440 (62.9%) |
|  | 25-35 years | 141 (20.1%) | Female | 254 (36.3%) | 10-20 years | 195 (27.9%) |
|  | 36-45 years | 309 (44.1%) | Diverse | 5 (0.7%) | >20 years | 65 (9.3%) |
|  | >45 years | 244 (34.9%) |  |  |  |  |
| USA (n = 222) | <25 years | 4 (1.8%) | Male | 152 (68.5%) | <10 years | 125 (56.3%) |
|  | 25-35 years | 36 (16.2%) | Female | 67 (30.2%) | 10-20 years | 47 (21.2%) |
|  | 36-45 years | 41 (18.5%) | Diverse | 3 (1.4%) | >20 years | 50 (22.5%) |
|  | >45 years | 141 (63.5%) |  |  |  |  |
| TOTAL (n = 1961) | <25 years | 33 (1.7%) | Male | 1287 (65.6%) | <10 years | 1017 (51.9%) |
|  | 25-35 years | 429 (21.9%) | Female | 659 (33.6%) | 10-20 years | 533 (27.2%) |
|  | 36-45 years | 705 (36.0%) | Diverse | 15 (0.8%) | >20 years | 411 (21.0%) |
|  | >45 years | 794 (40.5%) |  |  |  |  |

*Notes.* Survey overview based on all participants. Participants with diverse gender (n = 15) could not be considered in later analyses that were adjusted for gender.

**Table S3 (Supplement).** Categorical distribution of depression, anxiety, stress and insomnia

|  |  |  | **Sample without diverse gender** | |
| --- | --- | --- | --- | --- |
| **Continent** | **Variable** | **Category** | **n** | **%** |
| **Overall** |  |  |  |  |
|  | Depression | Normal | 1166 | 59.9 |
|  |  | Mild | 245 | 12.6 |
|  |  | Moderate | 312 | 16.0 |
|  |  | Severe | 109 | 5.6 |
|  |  | Extremely severe | 114 | 5.9 |
|  | Anxiety | Normal | 1322 | 67.9 |
|  |  | Mild | 124 | 6.4 |
|  |  | Moderate | 280 | 14.4 |
|  |  | Severe | 80 | 4.1 |
|  |  | Extremely severe | 140 | 7.2 |
|  | Stress | Normal | 1372 | 70.5 |
|  |  | Mild | 204 | 10.5 |
|  |  | Moderate | 200 | 10.3 |
|  |  | Severe | 132 | 6.8 |
|  |  | Extremely severe | 38 | 2.0 |
|  | Insomnia | Not clinically significant | 1002 | 51.5 |
|  |  | Subthreshold | 590 | 30.3 |
|  |  | Clinical, moderate | 298 | 15.3 |
|  |  | Clinical, severe | 56 | 2.9 |
| **Europe** |  |  |  |  |
|  | Depression | Normal | 584 | 56.6 |
|  |  | Mild | 137 | 13.3 |
|  |  | Moderate | 162 | 15.7 |
|  |  | Severe | 75 | 7.3 |
|  |  | Extremely severe | 74 | 7.2 |
|  | Anxiety | Normal | 726 | 70.3 |
|  |  | Mild | 62 | 6.0 |
|  |  | Moderate | 125 | 12.1 |
|  |  | Severe | 44 | 4.3 |
|  |  | Extremely severe | 75 | 7.3 |
|  | Stress | Normal | 680 | 65.9 |
|  |  | Mild | 106 | 10.3 |
|  |  | Moderate | 126 | 12.2 |
|  |  | Severe | 95 | 9.2 |
|  |  | Extremely severe | 25 | 2.4 |
|  | Insomnia | Not clinically significant | 509 | 49.3 |
|  |  | Subthreshold | 313 | 30.3 |
|  |  | Clinical, moderate | 175 | 17.0 |
|  |  | Clinical, severe | 35 | 3.4 |
| **Asia** |  |  |  |  |
|  | Depression | Normal | 434 | 62.4 |
|  |  | Mild | 87 | 12.5 |
|  |  | Moderate | 119 | 17.1 |
|  |  | Severe | 30 | 4.3 |
|  |  | Extremely severe | 25 | 3.6 |
|  | Anxiety | Normal | 424 | 61.0 |
|  |  | Mild | 54 | 7.8 |
|  |  | Moderate | 131 | 18.8 |
|  |  | Severe | 29 | 4.2 |
|  |  | Extremely severe | 57 | 8.2 |
|  | Stress | Normal | 529 | 76.1 |
|  |  | Mild | 72 | 10.4 |
|  |  | Moderate | 57 | 8.2 |
|  |  | Severe | 28 | 4.0 |
|  |  | Extremely severe | 9 | 1.3 |
|  | Insomnia | Not clinically significant | 373 | 53.7 |
|  |  | Subthreshold | 212 | 30.5 |
|  |  | Clinical, moderate | 95 | 13.7 |
|  |  | Clinical, severe | 15 | 2.2 |
| **USA** |  |  |  |  |
|  | Depression | Normal | 148 | 67.6 |
|  |  | Mild | 21 | 9.6 |
|  |  | Moderate | 31 | 14.2 |
|  |  | Severe | 4 | 1.8 |
|  |  | Extremely severe | 15 | 6.8 |
|  | Anxiety | Normal | 172 | 78.5 |
|  |  | Mild | 8 | 3.7 |
|  |  | Moderate | 24 | 11.0 |
|  |  | Severe | 7 | 3.2 |
|  |  | Extremely severe | 8 | 3.7 |
|  | Stress | Normal | 163 | 74.4 |
|  |  | Mild | 26 | 11.9 |
|  |  | Moderate | 17 | 7.8 |
|  |  | Severe | 9 | 4.1 |
|  |  | Extremely severe | 4 | 1.8 |
|  | Insomnia | Not clinically significant | 120 | 54.8 |
|  |  | Subthreshold | 65 | 29.7 |
|  |  | Clinical, moderate | 28 | 12.8 |
|  |  | Clinical, severe | 6 | 2.7 |

*Notes.* Category ranges based on scores, no transformations applied. Categories assumed as follows: Depression: 0-9 = normal, 10-13 = mild, 14-20 = moderate, 21-27 = severe, 28+ = extremely severe. Anxiety: 0-7 = normal, 8-9 = mild, 10-14 = moderate, 15-19 = severe, 20+ = extremely severe. Stress: 0-14 = normal, 15-18 = mild, 19-25 = moderate, 26-33 = severe, 34+ = extremely severe. Insomnia: 0-7 = no clinically significant insomnia, 8-14 = subthreshold insomnia, 15-21 = clinical insomnia (moderate severity), 22+ = clinical insomnia (severe).

**Table S4 (Supplement).** Means [95% confidence interval] adjusted for age and gender for Europe, Asia and the USA, p-value for comparison of continents and corresponding effect size

|  | Europe (n=1032) | Asia (n=685) | USA (n=219) | p-value | Effect size (partial eta²) |
| --- | --- | --- | --- | --- | --- |
| Self-efficacy (GSE) | 4.24 [4.16-4.32] | 3.94 [3.85-4.02] | 4.29 [4.19-4.43] | <0.001 | 0.099 |
| Physical health (SF-12) | 50.87 [52.07-49.63] | 49.77 [51.06-48.45] | 52.62 [54.02-51.18] | <0.001 | 0.015 |
| Mental health (SF-12) | 47.36 [49.10-45.57] | 47.56 [49.37-45.69] | 46.50 [48.62-44.30] | 0.189 | 0.002 |
| Well-being (WHO-5) | 52.76 [48.89-56.63] | 61.47 [57.43-65.51] | 55.74 [51.09-60.39] | <0.001 | 0.032 |
| Depression (DASS-21) | 8.50 [6.64-10.32] | 6.45 [4.87-8.39] | 6.14 [4.42-8.38] | <0.001 | 0.010 |
| Anxiety (DASS-21) | 4.43 [3.40-5.65] | 6.39 [5.04-7.96] | 3.50 [2.39-4.81] | <0.001 | 0.020 |
| Stress (DASS-21) | 11.65 [9.94-13.28] | 9.17 [7.53-10.91] | 9.81 [7.87-11.85] | <0.001 | 0.015 |
| Insomnia (ISI) | 6.03 [5.05-7.21] | 5.04 [4.18-6.07] | 5.42 [4.38-6.72] | <0.001 | 0.008 |

**Table S5 (Supplement).** Adjusted regression coefficient [95% confidence interval] and p-values (below) for the predictors with respect to self-efficacy and the other dependent variables

| Predictor | Reference | Self-efficacy (GSE) | Physical health (SF;12) | Mental health (SF;12) | Well-being (WHO-5) | Depression (DASS-21) | Anxiety (DASS-21) | Stress (DASS-21) | Insomnia (ISI) |
| --- | --- | --- | --- | --- | --- | --- | --- | --- | --- |
| Partner | w/o | 0.041 [-0.073;0.156]  0.482 | -0.003 [-0.011;0.004]  0.405 | 0.002 [-0.008;0.013]  0.671 | 1.307 [-1.494;4.108]  0.360 | -0.077 [-0.194;0.040]  0.198 | 0.035 [-0.078;0.148]  0.539 | 0.002 [-0.119;0.124]  0.972 | 0.018 [-0.038;0.074]  0.533 |
| Children | w/o | 0.053 [-0.037;0.143]  0.246 | 0.002 [-0.004;0.008]  0.528 | 0.002 [-0.006;0.010]  0.640 | 0.644 [-1.549;2.837]  0.565 | -0.082 [-0.173;0.010]  0.082 | -0.034 [-0.123;0.054]  0.449 | -0.012 [-0.107;0.083]  0.806 | -0.028 [-0.072;0.016]  0.208 |
|  |  |  |  |  |  |  |  |  |  |
| Education >12 y | 0-9 y | 0.108 [-0.031;0.247]  0.128 | 0.027 [0.018;0.037]  <0.001 | 0.002 [-0.011;0.014]  0.815 | 0.155 [-3.244;3.553]  0.929 | -0.102 [-0.244;0.041]  0.162 | -0.169 [-0.306;-0.032]  0.016 | -0.073 [-0.221;0.074]  0.330 | -0.031 [-0.099;0.036]  0.365 |
| Education 10-12 y | 0-9 y | 0.081 [-0.089;0.251]  0.351 | 0.009 [-0.002;0.020]  0.125 | -0.005 [-0.021;0.010]  0.493 | -0.910 [-5.068;3.248]  0.668 | 0.110 [-0.064;0.284]  0.216 | -0.021 [-0.189;0.146]  0.802 | -0.016 [-0.197;0.165]  0.863 | 0.052 [-0.031;0.135]  0.218 |
|  |  |  |  |  |  |  |  |  |  |
| Employment >20 y | <10 y | -0.007 [-0.130;0.116]  0.910 | -0.006 [-0.014;0.002]  0.143 | -0.006 [-0.017;0.005]  0.295 | -3.060 [-6.064;-0.056]  0.046 | 0.110 [-0.015;0.236]  0.086 | 0.167 [0.046;0.288]  0.007 | 0.075 [-0.056;0.205]  0.263 | 0.065 [0.005;0.125]  0.035 |
| Employment 10-20 y | <10 y | 0.013 [-0.085;0.112]  0.790 | -0.002 [-0.009;0.004]  0.481 | -0.003 [-0.012;0.006]  0.514 | -2.277 [-4.681;0.128]  0.064 | 0.083 [-0.018;0.183]  0.108 | 0.048 [-0.049;0.145]  0.333 | 0.062 [-0.043;0.166]  0.246 | 0.007 [-0.041;0.055]  0.784 |
|  |  |  |  |  |  |  |  |  |  |
| Executive | No | 0.139 [0.049;0.229]  0.003 | -0.001 [-0.007;0.005]  0.848 | 0.015 [0.007;0.023]  <0.001 | 4.005 [1.800;6.210]  <0.001 | -0.164 [-0.256;-0.072]  0.001 | -0.124 [-0.213;-0.036]  0.006 | -0.119 [-0.215;-0.024]  0.015 | -0.044 [-0.088;0.000]  0.052 |
|  |  |  |  |  |  |  |  |  |  |
| Home office >75% | No | 0.131 [0.021;0.241]  0.019 | 0.021 [0.014;0.028]  <0.001 | 0.015 [0.005;0.025]  0.005 | 4.683 [1.999;7.367]  0.001 | -0.214 [-0.326;-0.102]  <0.001 | -0.262 [-0.371;-0.154]  <0.001 | -0.239 [-0.356;-0.123]  <0.001 | -0.056 [-0.110;-0.002]  0.041 |
| Home office >50% | No | 0.037 [-0.107;0.181]  0.614 | 0.022 [0.012;0.031]  <0.001 | 0.006 [-0.008;0.019]  0.411 | 2.418 [-1.104;5.940]  0.178 | -0.147 [-0.295;0.000]  0.050 | -0.258 [-0.400;-0.116]  <0.001 | -0.189 [-0.342;-0.036]  0.015 | -0.026 [-0.096;0.044]  0.470 |
| Home office >25% | No | 0.050 [-0.086;0.186]  0.472 | 0.020 [0.011;0.029]  <0.001 | 0.015 [0.002;0.027]  0.021 | 3.993 [0.673;7.312]  0.018 | -0.183 [-0.321;-0.044]  0.010 | -0.183 [-0.317;-0.049]  0.007 | -0.221 [-0.365;-0.076]  0.003 | -0.050 [-0.116;0.016]  0.141 |
|  |  |  |  |  |  |  |  |  |  |
| USA | Europe | 0.076 [-0.058;0.211]  0.265 | 0.010 [0.001;0.019]  0.022 | -0.011 [-0.024;0.001]  0.074 | 1.714 [-1.565;4.994]  0.306 | -0.131 [-0.268;0.006]  0.061 | -0.063 [-0.195;0.069]  0.349 | -0.131 [-0.273;0.012]  0.072 | -0.013 [-0.079;0.052]  0.692 |
| Asia | Europe | -0.630 [-0.727;-0.534]  <0.001 | -0.017 [-0.024;-0.011]  <0.001 | -0.004 [-0.013;0.004]  0.327 | 6.744 [4.387;9.102]  <0.001 | -0.067 [-0.166;0.032]  0.183 | 0.362 [0.267;0.457]  <0.001 | -0.168 [-0.270;-0.065]  0.001 | -0.044 [-0.091;0.004]  0.070 |

**Table S6 (Supplement).** Standardized regression coefficients [95% confidence interval] and p-values for demographic and other predictors including psychological scales with respect to different aspects of satisfaction overall and for aspects of satisfaction with COVID-19 management

| Predictor | Reference | work satisfaction | COVID-19  info satisfaction | COVID-19  managmt. satisf. | feeling protected | sufficient equipmnt. | managmt. support |
| --- | --- | --- | --- | --- | --- | --- | --- |
| Partner | No | 0.74 [2.70;-1.22]  0.461 | 0.46 [-1.50;2.42]  0.647 | -1.31 [-3.27;0.65]  0.191 | -2.16 [-4.12;-0.20]  0.031 | -1.80 [-3.76;0.16]  0.072 | -0.18 [-2.14;1.78]  0.854 |
|  |  |  |  |  |  |  |  |
| Children | No | -1.37 [0.59;-3.33]  0.170 | -1.01 [-2.97;0.95]  0.314 | 1.09 [-0.87;3.05]  0.277 | -0.90 [-2.86;1.06]  0.370 | -0.91 [-2.87;1.05]  0.362 | -1.57 [-3.53;0.39]  0.116 |
|  |  |  |  |  |  |  |  |
| Education >12 y | 0-9 y | 1.34 [3.30;-0.62]  0.182 | 1.47 [-0.49;3.43]  0.142 | -0.58 [-2.54;1.38]  0.559 | 0.67 [-1.29;2.63]  0.505 | 2.40 [0.44;4.36]  0.016 | 0.20 [-1.76;2.16]  0.843 |
| Education 10-12 y | 0-9 y | 0.57 [2.53;-1.39]  0.571 | 1.52 [-0.44;3.48]  0.130 | 0.42 [-1.54;2.38]  0.674 | -0.47 [-2.43;1.49]  0.636 | 1.60 [-0.36;3.56]  0.109 | 1.28 [-0.68;3.24]  0.199 |
|  |  |  |  |  |  |  |  |
| Employment >20 y | <10 y | 0.88 [2.84;-1.08]  0.379 | 1.45 [-0.51;3.41]  0.146 | -0.10 [-2.06;1.86]  0.919 | 2.29 [0.33;4.25]  0.022 | 1.02 [-0.94;2.98]  0.307 | 2.02 [0.06;3.98]  0.044 |
| Employment 10-20 y | <10 y | 0.59 [2.55;-1.37]  0.558 | 0.78 [-1.18;2.74]  0.435 | 0.05 [-1.91;2.01]  0.958 | 0.87 [-1.09;2.83]  0.382 | -0.80 [-2.76;1.16]  0.426 | 0.67 [-1.29;2.63]  0.502 |
|  |  |  |  |  |  |  |  |
| Home office >75% | No | 2.80 [4.76;0.84]  0.005 | -1.77 [-3.73;0.19]  0.078 | -8.71 [-10.67;-6.75]  <0.001 | -1.45 [-3.41;0.51]  0.148 | 2.01 [0.05;3.97]  0.045 | -4.10 [-6.06;-2.14]  <0.001 |
| Home office >50% | No | 1.65 [3.61;-0.31]  0.099 | -2.05 [-4.01;-0.09]  0.041 | -4.74 [-6.70;-2.78]  <0.001 | 0.28 [-1.68;2.24]  0.777 | 2.22 [0.26;4.18]  0.027 | -2.16 [-4.12;-0.20]  0.031 |
| Home office >25% | No | 2.85 [4.81;0.89]  0.004 | -2.33 [-4.29;-0.37]  0.020 | -4.31 [-6.27;-2.35]  <0.001 | -0.56 [-2.52;1.40]  0.577 | 0.67 [-1.29;2.63]  0.504 | -1.74 [-3.70;0.22]  0.082 |
|  |  |  |  |  |  |  |  |
| Executive | No | 1.04 [3.00;-0.92]  0.299 | 1.03 [-0.93;2.99]  0.304 | -0.51 [-2.47;1.45]  0.608 | -3.33 [-5.28;-1.37]  <0.001 | -3.00 [-4.96;-1.04]  0.003 | -0.07 [-2.03;1.89]  0.942 |
|  |  |  |  |  |  |  |  |
| USA | Europe | 3.25 [5.21;1.29]  0.001 | -0.13 [-2.09;1.83]  0.893 | 4.65 [2.69;6.61]  <0.001 | 5.31 [3.35;7.27]  <0.001 | 6.84 [4.88;8.80]  <0.001 | 0.06 [-1.90;2.02]  0.955 |
| Asia | Europe | 3.79 [5.75;1.83]  <0.001 | -3.75 [-5.71;-1.79]  <0.001 | 0.81 [-1.15;2.77]  0.419 | 8.29 [6.33;10.25]  <0.001 | 7.19 [5.23;9.15]  <0.001 | 2.68 [0.72;4.64]  0.007 |
|  |  |  |  |  |  |  |  |
| Infection | No |  | -0.02 [-1.98;1.94]  0.981 | -0.69 [-2.65;1.27]  0.492 | 0.35 [-1.61;2.31]  0.724 | 0.65 [-1.31;2.61]  0.517 | 0.28 [-1.68;2.24]  0.780 |
| Death | No |  | -0.08 [-2.04;1.88]  0.937 | -0.59 [-2.55;1.37]  0.556 | 0.33 [-1.63;2.29]  0.744 | 1.86 [-0.10;3.82]  0.063 | -0.51 [-2.47;1.45]  0.611 |
|  |  |  |  |  |  |  |  |
| Well-being (WHO-5) |  | -7.57 [-5.61;-9.53]  <0.001 | -6.51 [-8.47;-4.55]  <0.001 | -4.36 [-6.32;-2.40]  <0.001 | -1.70 [-3.66;0.26]  0.088 | 0.04 [-1.92;2.00]  0.965 | -2.81 [-4.77;-0.85]  0.005 |
| Depression (DASS-21) |  | 5.11 [7.07;3.15]  <0.001 | 2.81 [0.85;4.77]  0.005 | 3.05 [1.09;5.01]  0.002 | 1.40 [-0.56;3.36]  0.161 | -0.61 [-2.57;1.35]  0.544 | 2.57 [0.61;4.53]  0.010 |
| Stress (DASS-21) |  | -0.04 [1.92;-2.00]  0.965 | 1.67 [-0.29;3.63]  0.094 | -2.29 [-4.25;-0.33]  0.022 | -1.27 [-3.23;0.69]  0.205 | -0.37 [-2.33;1.59]  0.711 | -0.61 [-2.57;1.35]  0.539 |
| Anxiety (DASS-21) |  | -2.40 [-0.44;-4.36]  0.016 | -2.38 [-4.34;-0.42]  0.017 | -0.48 [-2.44;1.48]  0.630 | 0.15 [-1.81;2.11]  0.882 | 1.70 [-0.26;3.66]  0.089 | -0.09 [-2.05;1.87]  0.931 |
| Insomnia (ISI) |  | 2.00 [3.96;0.04]  0.046 | 2.55 [0.59;4.51]  0.011 | 0.83 [-1.13;2.79]  0.405 | 2.04 [0.08;4.00]  0.042 | 1.03 [-0.93;2.99]  0.305 | 3.30 [1.34;5.26]  <0.001 |
| Physical health  (SF-12) |  | -2.08 [-4.04;-0.12]  0.037 | -1.47 [0.49;-3.43]  0.142 | -3.81 [-1.85;-5.77]  <0.001 | -5.07 [-3.11;-7.03]  <0.001 | -5.41 [-3.45;-7.37]  <0.001 | -4.04 [-2.08;-6.00]  <0.001 |
| Mental health  (SF-12) |  | -4.48 [-6.44;-2.52]  <0.001 | -1.15 [0.81;-3.11]  0.251 | -3.49 [-1.53;-5.45]  <0.001 | -3.04 [-1.08;-5.00]  0.002 | -2.74 [-0.78;-4.70]  0.006 | -2.17 [-0.21;-4.13]  0.030 |
| Self-efficacy (GSE) |  | -4.72 [-2.76;-6.68]  <0.001 | -1.27 [-3.23;0.69]  0.204 | -1.25 [-3.21;0.71]  0.213 | -2.02 [-3.98;-0.06]  0.043 | -1.87 [-3.83;0.09]  0.061 | -0.66 [-2.62;1.30]  0.507 |

**Table S7 (Supplement).** Differences in comparison of high-response countries (mostly Austria) and low-response countries in Europe

|  |  | Low-governmental response Europe | | High-governmental response Europe | |  |  |
| --- | --- | --- | --- | --- | --- | --- | --- |
| Sample | Variable | Median (IQR) | Mean Rank | Median (IQR) | Mean Rank | U | p* |
| overall |  | (n =682) | | (n =350) | |  |  |
|  | COVID-19 info satisfaction  (1=very satisfied; 5=very unsatisfied) | 3 (2-4) | 530.21 | 3 (2-3) | 489.78 | 109999.0 | 0.032 |
|  | COVID-19 management  (1=very satisfied; 5=very unsatisfied) | 2 (1-3) | 506.93 | 2 (1-3) | 535.15 | 112821.5 | 0.131 |
|  | Feeling protected  (1=yes, 5=no) | 1 (1-2) | 531.11 | 1 (1-2) | 488.04 | 109388.5 | 0.009 |
|  | Fear of COVID-19  (1=yes, 5=no) | 4 (3-5) | 489.29 | 4 (3-5) | 569.52 | 100794.0 | <0.001 |
|  | Danger of COVID-19  (1=yes, 5=no) | 4 (3-4) | 490.83 | 4 (3-5) | 566.52 | 101844.8 | <0.001 |
|  | Self-efficacy (score) | 4 (4-4.67) | 505.14 | 4.33 (4-4.67) | 538.63 | 111604.0 | 0.079 |
|  | Well-being (score) | 56 (36-72) | 508.94 | 56 (36-72) | 531.23 | 114195.5 | 0.255 |
|  | Depression (score) | 8 (2-14) | 522.96 | 6 (2-16) | 503.91 | 114942.5 | 0.328 |
|  | Stress (score) | 10 (6-18) | 520.71 | 10 (4-18) | 508.30 | 116481.0 | 0.526 |
|  | Anxiety (score) | 2 (0-8) | 507.78 | 2 (0-10) | 533.48 | 113406.0 | 0.178 |
|  | Insomnia (score) | 8 (4-13) | 516.91 | 7 (4-14) | 515.70 | 119070.5 | 0.951 |
|  | Physical health quality of life (score) | 53.49 (47.70-55.70) | 528.65 | 52.22 (46.21-55.50) | 492.82 | 111062.0 | 0.067 |
|  | Mental health quality of life (score) | 50.35 (39.79-55.92) | 506.83 | 50.87 (39.95-57.15) | 535.34 | 112757.0 | 0.146 |
| Age <= 35 |  | (n =156) | | (n =117) | |  |  |
|  | COVID-19 info satisfaction |  | 140.11 |  | 132.85 | 8641.0 | 0.436 |
|  | COVID-19 management |  | 128.53 |  | 148.29 | 7805.0 | 0.034 |
|  | Feeling protected |  | 142.18 |  | 130.09 | 8318.0 | 0.124 |
|  | Fear of COVID-19 |  | 120.58 |  | 158.90 | 6564.0 | <0.001 |
|  | Danger of COVID-19 |  | 121.07 |  | 158.24 | 6641.0 | <0.001 |
|  | Self-efficacy |  | 130.18 |  | 146.09 | 8062.0 | 0.090 |
|  | Well-being |  | 141.59 |  | 130.88 | 8409.5 | 0.266 |
|  | Depression |  | 134.18 |  | 140.76 | 8686.0 | 0.494 |
|  | Stress |  | 132.65 |  | 142.79 | 8448.0 | 0.494 |
|  | Anxiety |  | 133.09 |  | 142.22 | 8515.5 | 0.338 |
|  | Insomnia |  | 138.64 |  | 134.81 | 8869.5 | 0.691 |
|  | Physical health quality of life |  | 139.39 |  | 133.88 | 8761.0 | 0.572 |
|  | Mental health quality of life |  | 139.88 |  | 133.15 | 8676.0 | 0.486 |
| Age 36-45 |  | (n =263) | | (n =89) | |  |  |
|  | COVID-19 info satisfaction |  | 180.93 |  | 163.42 | 10539.5 | 0.143 |
|  | COVID-19 management |  | 180.59 |  | 164.42 | 10628.0 | 0.173 |
|  | Feeling protected |  | 176.95 |  | 175.17 | 11585.5 | 0.867 |
|  | Fear of COVID-19 |  | 171.88 |  | 190.15 | 10489.0 | 0.133 |
|  | Danger of COVID-19 |  | 175.37 |  | 179.83 | 11407.5 | 0.713 |
|  | Self-efficacy |  | 178.51 |  | 170.57 | 11175.5 | 0.512 |
|  | Well-being |  | 167.68 |  | 202.56 | 9384.0 | 0.005 |
|  | Depression |  | 185.33 |  | 150.39 | 9380.0 | 0.005 |
|  | Stress |  | 182.38 |  | 159.12 | 10157.0 | 0.062 |
|  | Anxiety |  | 178.48 |  | 170.65 | 11183.0 | 0.518 |
|  | Insomnia |  | 178.87 |  | 169.48 | 11079.0 | 0.451 |
|  | Physical health quality of life |  | 178.84 |  | 169.58 | 11088.0 | 0.458 |
|  | Mental health quality of life |  | 167.78 |  | 202.27 | 9410.0 | 0.006 |
| Age: 45+ |  | (n =263) | | (n =144) | |  |  |
|  | COVID-19 info satisfaction |  | 210.27 |  | 192.55 | 17287.0 | 0.129 |
|  | COVID-19 management |  | 200.28 |  | 210.80 | 17957.5 | 0.358 |
|  | Feeling protected |  | 212.99 |  | 187.58 | 16572.0 | 0.013 |
|  | Fear of COVID-19 |  | 199.71 |  | 211.83 | 17808.5 | 0.308 |
|  | Danger of COVID-19 |  | 195.86 |  | 218.86 | 16796.0 | 0.052 |
|  | Self-efficacy |  | 196.38 |  | 217.92 | 16931.5 | 0.070 |
|  | Well-being |  | 201.02 |  | 209.45 | 18151.0 | 0.488 |
|  | Depression |  | 205.76 |  | 200.79 | 18473.5 | 0.681 |
|  | Stress |  | 207.01 |  | 198.51 | 18145.0 | 0.484 |
|  | Anxiety |  | 199.54 |  | 212.14 | 17763.5 | 0.284 |
|  | Insomnia |  | 202.42 |  | 206.89 | 18519.5 | 0.713 |
|  | Physical health quality of life |  | 212.01 |  | 189.38 | 16830.0 | 0.063 |
|  | Mental health quality of life |  | 198.95 |  | 213.22 | 17609.0 | 0.242 |

*Notes.* Sample based on male and female participants. Quartiles based on empirical percentile method. *p-values uncorrected, asympt. 2-sided. No transformations of scores applied.

## Supplementary Figures


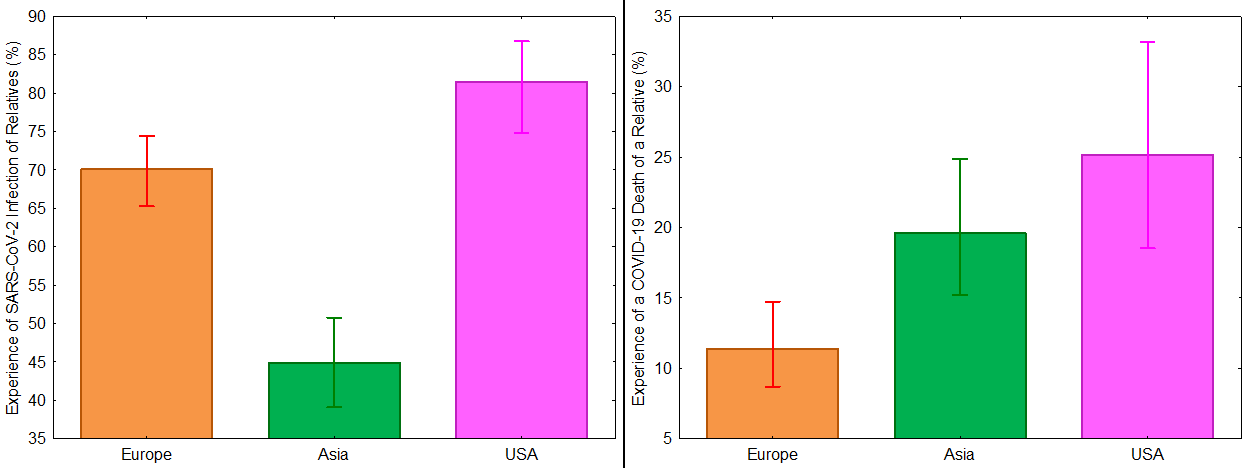


**Supplementary Figure S1.** Graphical illustration of personal experience with COVID-19-infections or –deaths. Whiskers represent the 95% confidence interval.


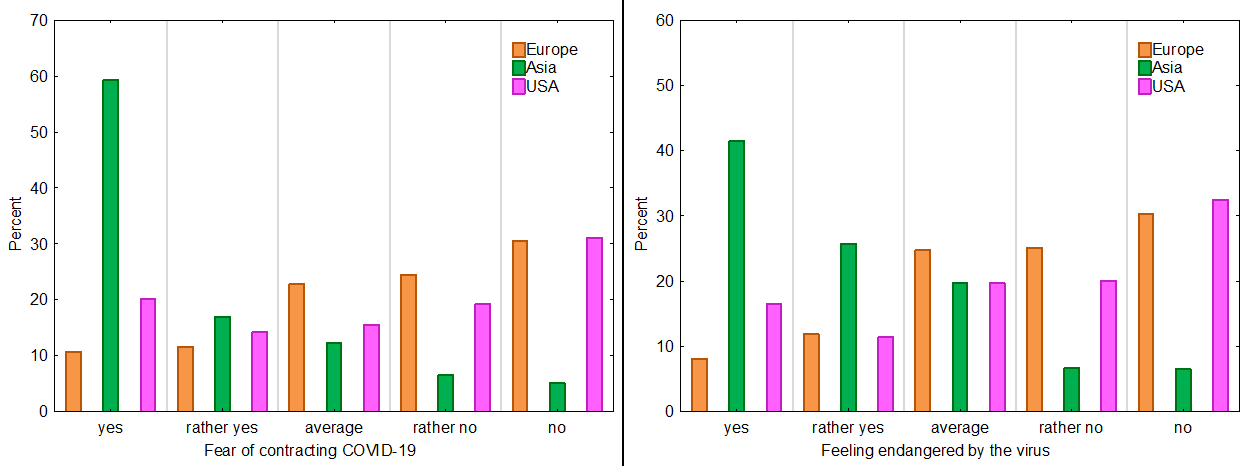


**Supplementary Figure S2.** Continent-specific differences regarding fear of contracting COVID-19 (left) and feeling endangered by the virus (right).
